# Current GMP standards for the large‐scale production of monoclonal antibodies

**DOI:** 10.1002/btm2.70121

**Published:** 2026-02-09

**Authors:** Tays Troncoso‐Bravo, Valentina Pavez, Pedro Letelier, Javier Del Río, Cristian Anabalón, Hernán F. Peñaloza, Pablo A. González, Susan M. Bueno, Alexis M. Kalergis

**Affiliations:** ^1^ Millennium Institute on Immunology and Immunotherapy, Facultad de Ciencias Biológicas Pontificia Universidad Católica de Chile Santiago Chile; ^2^ Química y Farmacia, Facultad de Química y Biología Universidad de Santiago de Chile Santiago Chile; ^3^ Departamento de Laboratorios Clínicos, Facultad de Medicina Pontificia Universidad Católica de Chile Santiago Chile; ^4^ Departamento de Endocrinología, Facultad de Medicina Pontificia Universidad Católica de Chile Santiago Chile

**Keywords:** bioprocesses, good manufacturing practices (GMPs), monoclonal antibodies (mAbs), pharmaceutical regulation, quality control

## Abstract

Monoclonal antibodies have revolutionized modern medicine due to their target‐specific properties and effectiveness in treating a wide range of diseases, including cancer, autoimmune disorders, infectious diseases, and neurological conditions. Importantly, their large‐scale production for human use requires strict adherence to good manufacturing practice (GMP) standards to ensure quality, safety, and efficacy. This article reviews key aspects of monoclonal antibody production under GMP standards, from cell‐line selection to culture strategies, antibody purification, formulation, and quality control processes. Additionally, we discuss the significance of validation and traceability in production, as well as the implementation of emerging technologies to enhance manufacturing efficiency and safety. Despite progress in bioprocesses and regulatory frameworks, several challenges, such as batch‐to‐batch variability, high production costs, and the need to continuously adapt processes to new regulations, remain to be solved. The integration of innovative approaches with evolving regulations will enable the optimization of monoclonal antibody production and ensure their global accessibility.

## Graphical abstract

**Production of monoclonal antibodies (mAbs) under good manufacturing practices (GMP)**. mAbs are uniform biological molecules derived from a single B‐cell clone, renowned for their high specificity and widely employed as therapeutic and diagnostic agents. The development process for mAbs begins with the design and selection of antibodies (Abs), utilizing techniques such as hybridoma technology or phage display systems. Once the appropriate Abs are identified, their genes are expressed in recombinant mammalian cell lines, which typically include Chinese hamster ovary cells, human embryonic kidney cells, or murine myeloma (NS0) cells. The production of mAbs on a large scale takes place in bioreactors operated in batch, fed batch, or continuous modes. During this phase, key parameters—including pH, dissolved oxygen levels, osmolality, and nutrient concentrations—are closely monitored to ensure optimal conditions are maintained. The subsequent downstream process begins with purification through affinity chromatography, frequently employing Protein A from *Staphylococcus aureus* or Protein G from *Streptococcus* spp. This purification step is followed by ultrafiltration and diafiltration to eliminate impurities and concentrate the final product. After purification, the Abs are formulated and stored in vials under refrigerated conditions (2–8°C) or as lyophilized powder to ensure their long‐term stability. Throughout the manufacturing process, robust validation and traceability systems are essential. These systems utilize tools such as blockchain technology, quick response codes, radio frequency identification tags, and Internet of Things sensors to maintain the transparency and integrity of the data. A comprehensive quality control framework, guided by World Health Organization and European Medicines Agency standards, encompasses physical, chemical, biological, and microbiological testing. This framework ensures compliance with quality by design principles, effective control of process parameters, and adherence to critical quality attributes. By implementing GMP standards, the consistent and safe production of mAbs is achieved, facilitating their use in a diverse array of clinical applications, including treatments for cancer, autoimmune disorders, gastrointestinal issues, and infectious diseases.

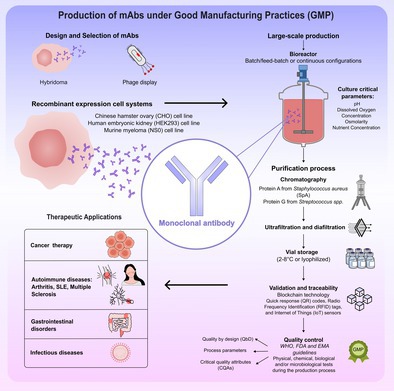

**Abbreviations**
Abs: antibodies
AD: Alzheimer's disease
ADCC: antibody‐dependent cellular cytotoxicity
ADCs: antibody–drug conjugates
ADLib: autonomous diversifying library
aHUS: atypical hemolytic uremic syndrome
ALCOA: attributable, legible, contemporaneous, original, and accurate
ANID: Agencia Nacional de Investigación y Desarrollo
anti‐TNF‐α: antitumor necrosis factor alpha
API: active pharmaceutical ingredient
BAFF: B‐cell activating factor
bNAbs: broadly neutralizing antibodies
CDMO: contract development and manufacturing organization
CDR: complementarity‐determining region
cFAE: confluent follicle‐associated epithelium
CGRP: calcitonin gene‐related peptide
CHO: Chinese hamster ovary
CLL: chronic lymphocytic leukemia
COVID‐19: Coronavirus Disease 2019
CQAs: critical quality attributes
CRISPR: clustered regularly interspaced short palindromic repeats
CRSwNP: chronic rhinosinusitis with nasal polyps
DHFR: dihydrofolate reductase
ELISAs: enzyme‐linked immunosorbent assays
EMA: European Medicines Agencies
F: fusion protein
FDA: Food and Drug Administration
GFC: gel filtration chromatography
gMG: generalized myasthenia gravis
GMPs: good manufacturing practices
GS: glutamine synthase
HAT: hypoxanthine–aminopterin–thymidine
Hc: heavy chain
HEK293: human embryonic kidney
HIC: hydrophobic interaction chromatography
HIV: human immunodeficiency virus
HPLC: high‐pressure liquid chromatography
hRSV: human respiratory syncytial virus
HVAC: heating, ventilation, and air conditioning
IBD: inflammatory bowel disease
ICH: International Council for Harmonisation
ICIs: immune checkpoint inhibitors
Igs: immunoglobulins
IL‐6: interleukin 6
IoT: Internet of Things
ISO: International Organization for Standardization
Lc: light chain
LDL: low‐density lipoprotein
mAbs: monoclonal antibodies
MG: myasthenia gravis
MS: multiple sclerosis
NMR: nuclear magnetic resonance
NSCLC: non‐small cell lung cancer
ORF: open reading frame
PD‐1: programmed death‐1
PD‐L1: programmed cell death ligand 1
PPQ: process performance qualification
PQS: pharmaceutical quality system
QbD: quality by design
QC: quality control
QR: quick response
R&D: research and development
RFID: radio frequency identification
RT‐PCR: reverse transcription polymerase chain reaction
SLE: systemic lupus erythematosus
SpA: *Staphylococcus aureus*
SPR: surface plasmon resonance
T1D: type 1 diabetes
TNBC: triple‐negative breast cancer
Trop‐2: trophoblast cell surface antigen 2
TSA: trichostatin A
TSLP: thymic stromal lymphopoietin
VHH: variable domain of heavy‐chain‐only antibodies
VSCC: vulvar squamous cell carcinoma

**Translational Impact Statement**

This review provides an overview of current good manufacturing practice standards and emerging technologies in the large‐scale production of monoclonal antibodies (mAbs). It emphasizes strategies designed to enhance product quality, ensure consistency, and maintain regulatory compliance. By addressing key production challenges such as scalability, cost efficiency, and traceability, this study informs the advancement of more effective biomanufacturing processes, ultimately enhancing clinical access to safe and effective mAbs therapies.

## 1 INTRODUCTION

The production of biologicals for medical use presents a complex challenge due to the high regulatory and production standards required to ensure the quality of the final product.^1^ Good manufacturing practice (GMP) represents the gold standard for producing biologicals, guaranteeing quality, safety, and efficacy.^2^ Advanced therapeutic medicinal products, which include gene therapies, antibodies (Abs), cell‐based therapies, and tissue therapies, constitute a new category of biopharmaceuticals.^1^, ^3^ These products enable the treatment of numerous diseases that previously had limited or ineffective therapeutic alternatives.^3^

Monoclonal antibodies (mAbs) are uniform biological molecules produced by a single B‐cell clone, making them powerful and innovative tools for developing therapies and diagnostic applications.^4^ These applications have recently garnered significant attention from the medical and pharmaceutical communities, establishing mAbs as a substantial component of the biopharmaceutical market.^1^, ^4^

This review builds on our initial exploration of current GMP vaccine and antibody production standards, which explicitly focus on mAbs.^5^ In this article, we discuss the key guidelines and regulations governing mAb production, address the challenges encountered during manufacturing, and assess essential aspects of quality control (QC), providing a comprehensive overview of the evolving landscape in this field.

## 2 MONOCLONAL ANTIBODIES AND THEIR THERAPEUTIC APPLICATIONS

Abs, also called immunoglobulins (Igs), are glycoproteins produced by B‐cell‐derived plasma cells.^6^ They are produced when the immune system elicits a response against one specific molecule or antigen. Physiologically, their primary purpose is to specifically bind to it and act as a line of defense against foreign, harmful bodies, such as pathogens.^7^

mAbs are molecules that are produced by homologous B cells that are derived from the cloning of a single parental cell and have a defined specificity.^8^ They can bind to a specific epitope or antigen and perform other effector functions.^8^ In recent years, mAbs have provided precise and targeted therapeutic strategies across various disease domains.^9^ Their ability to selectively bind antigens, modulate immune responses, and carry out direct cytotoxic effects has made them indispensable in oncology, autoimmune disorders, infectious diseases, neurological conditions, and gastroenterology, among other fields.^10^

Since mAbs used in therapies must have sequences of human origin or as similar as possible to human Igs, their development and manufacturing require high standards of quality and precision.^11^ Advances in antibody engineering, such as the use of antibody–drug conjugates (ADCs) and immune checkpoint inhibitors (ICIs), have further expanded their clinical utility, optimizing their efficacy and safety.^12^ This section examines the diverse applications of mAbs in various types of diseases, detailing their mechanisms and clinical implications.

### 2.1 Cancer

In modern oncology, mAbs target tumor‐specific antigens, modulate immune checkpoints, deliver cytotoxic agents to malignant cells, minimize off‐target toxicity, and enhance antitumor immunity.^13^ A prominent target is trophoblast cell surface antigen 2 (Trop‐2), a glycoprotein that is overexpressed in numerous cancers, including breast and vulvar squamous cell carcinoma (VSCC).^14^ Recent studies indicate that Trop‐2 expression is present in 97.1% of VSCC patients, suggesting that Trop‐2‐directed ADCs, such as sacituzumab govitecan, could offer effective therapeutic options for this type of cancer.^14^, ^15^

Sacituzumab govitecan, which links an anti‐Trop‐2 antibody to the cytotoxic agent SN‐38, has shown significant efficacy in triple‐negative breast cancer (TNBC), a particularly aggressive subtype with limited treatment options, and in patients with metastatic solid tumors (NCT03964727).^16^ In addition to TNBC, Trop‐2‐targeted therapies are being evaluated for their applicability across other epithelial malignancies.^17^

In addition to ADCs, ICIs, such as nivolumab and atezolizumab, which target the programmed death‐1 (PD‐1) receptor and programmed cell death ligand 1 (PD‐L1) on the surface of cancer cells, respectively, have transformed cancer immunotherapy by restoring or potentiating T‐cell activity against tumor cells.^18^ These therapies have shown clinical success in non‐small cell lung cancer (NSCLC), melanoma, and bladder cancer, significantly improving progression‐free periods and overall survival (NCT01844505 and NCT02008227).^19^

Other primary mAb‐based cancer therapies include rituximab (anti‐CD20) for B‐cell malignancies such as chronic lymphocytic leukemia (CLL) and non‐Hodgkin lymphoma, as well as trastuzumab (anti‐HER2) for HER2‐positive breast and gastric cancers.^20^ The integration of mAbs with small‐molecule inhibitors, radiation therapy, and chemotherapy continues to expand their therapeutic scope, increasing treatment efficacy across various malignancies.^21^

### 2.2 Autoimmune diseases

Autoimmune diseases arise from an overactive immune response that attacks self‐antigens, leading to chronic inflammation and organ damage.^22^ Unlike traditional immunosuppressants, mAbs offer a targeted approach for immune modulation, reducing the systemic side effects experienced by other drugs while improving therapeutic precision.^9^

Satralizumab, a humanized anti‐IL‐6 receptor mAb, has been evaluated for generalized myasthenia gravis (gMG).^23^ While a Phase 3 clinical trial (NCT04963270) confirmed its favorable safety profile, its clinical efficacy remains moderate, highlighting the need for further therapeutic optimization in gMG.^23^ Other mAbs that target autoimmunity‐related pathways include rituximab (anti‐CD20), which has shown efficacy in the treatment of rheumatoid arthritis, multiple sclerosis (MS), and systemic lupus erythematosus (SLE) by depleting B cells.^24^ Additionally, teplizumab (anti‐CD3) has shown promise in delaying type 1 diabetes (T1D) onset by modulating autoreactive T‐cell responses and preserving insulin‐producing beta cells.^25^ Established therapies such as tocilizumab (anti‐IL‐6) and belimumab (anti‐B‐cell activating factor [BAFF]) continue to play pivotal roles in SLE, juvenile idiopathic arthritis, and other autoimmune conditions.^26^

### 2.3 Infectious diseases

As infectious disease therapies, mAbs have contributed mainly to antiviral and antibacterial immunotherapies. One well‐characterized example is eculizumab, a complement C5 inhibitor used in atypical hemolytic uremic syndrome (aHUS)^27^ and severe COVID‐19.^28^ This mAb inhibits the complement cascade, preventing excessive downstream inflammation and complement‐mediated tissue damage.^27^, ^28^ Furthermore, broadly neutralizing antibodies (bNAbs), which target conserved viral epitopes to prevent immune escape and drug resistance, are being developed for human immunodeficiency virus (HIV) therapy.^29^

Moreover, the use of mAbs has emerged as a key strategy for immunoprophylaxis against human respiratory syncytial virus (hRSV), especially in high‐risk populations such as premature infants and immunocompromised patients.^30^ Palivizumab, a humanized mAb directed against the fusion protein (F) of hRSV, is one of the approved mAbs for such clinical use.^30^ Its mechanism of action is based on neutralizing the virus by inhibiting viral entry into host cells.^30^ However, its effectiveness has been shown to be moderate (approximately 55% in preventing hospitalizations). Palivizumab must be administered monthly, which represents a logistic limitation, and high cost restricts its application to highly susceptible populations.^30^, ^31^ Given these limitations, new‐generation mAbs with improved affinity and stability have been developed.^31^ Nirsevimab, recently approved by the Food and Drug Administration (FDA) for prophylactic use against hRSV, has a prolonged half‐life of up to 6 months due to modifications in its Fc region, allowing a single administration per season, with an efficacy of over 70% in preventing severe infections.^32^ Notably, this mAb neutralizes different strains of hRSV with >50 times greater activity than palivizumab does and protects infants against hospitalizations caused by hRSV infections in 82.4% of immunized patients.^33^, ^34^

Oligoclonal antibody approaches are also being explored for influenza, hRSV, and bacterial infections, offering a novel way to neutralize multiple pathogen strains simultaneously.^35^ Other investigational mAbs include tezepelumab (anti‐TSLP), which is currently under evaluation for chronic rhinosinusitis with nasal polyps (CRSwNP) and severe asthma (NCT03347279).^36^

### 2.4 Neurological disorders

mAbs are also reshaping treatment paradigms in neurology, particularly for migraines and neuroinflammatory disorders.^37^ Calcitonin gene‐related peptide (CGRP) inhibitors, such as erenumab (NCT02066415) and galcanezumab (NCT02614261), have demonstrated significant efficacy in reducing the frequency and severity of both chronic and/or episodic migraine by blocking CGRP, a key molecule in migraine pathophysiology.^38^

Another breakthrough in this area, specifically in neuroinflammatory disorders, is the development of FcRn‐targeting mAbs, such as efgartigimod (NCT03669588) and rozanolixizumab (NCT03971422).^39^ These mAbs enhance pathogenic autoantibody clearance in myasthenia gravis (MG), providing a highly targeted alternative to conventional immunosuppressants.^39^, ^40^ Similarly, in MS, anti‐CD20 mAbs such as ocrelizumab and ofatumumab selectively deplete B CD20^+^ cells, reducing disease progression and relapse rates.^37^

Emerging applications of mAbs in neurodegenerative diseases include their role in Alzheimer's disease (AD), with antiamyloid mAbs, such as aducanumab (NCT01677572) and lecanemab (NCT03887455), showing potential in reducing amyloid plaque burden.^41^ These therapies aim to slow cognitive decline by targeting beta‐amyloid aggregates, although their clinical efficacy remains a subject of ongoing debate.

### 2.5 Gastrointestinal disorders

In inflammatory bowel disease (IBD), mAbs have also been pivotal in targeting immune pathways that drive chronic inflammation.^42^ Antitumor necrosis factor alpha (anti‐TNF‐α) mAbs, such as infliximab (NCT00207662) and adalimumab (NCT00408629), were the first targeted therapies introduced for the treatment of moderate‐to‐severe IBD and have demonstrated efficacy in inducing and maintaining remission, reducing hospitalization rates, and promoting mucosal healing.^43^ Newer therapies, such as IL‐23p19 subunit‐specific mAbs, such as guselkumab and risankizumab, have shown promise because they selectively target the gut‐resident myeloid cells responsible for chronic inflammation.^44^

On the other hand, vedolizumab (an anti‐α4β7 integrin) selectively inhibits gut‐homing lymphocyte trafficking, reducing inflammation in patients with Crohn's disease and ulcerative colitis.^45^ Newer investigational therapies, such as the CCR9‐depleting mAb AZD7798, aim to block gut‐specific T‐cell migration, showing potential in the treatment of Crohn's disease.^46^

### 2.6 Emerging applications of mAbs

In addition to being explored for well‐established applications, mAbs are being explored for new therapeutic applications in multiple fields and diseases.^47^ In ocular diseases, anti‐VEGF mAbs such as ranibizumab (NCT00473330) and aflibercept (NCT04429503) have been instrumental in treating age‐related macular degeneration (AMD) and diabetic retinopathy, effectively inhibiting pathological angiogenesis and preserving vision.^48^

In metabolic and cardiovascular disorders, mAbs targeting PCSK9, a protein that regulates cholesterol levels, such as alirocumab (NCT01663402) and evolocumab (NCT03872401), have revolutionized the management of hypercholesterolemia.^49^ By enhancing LDL receptor recycling, these therapies effectively reduce cardiovascular risk.^49^

Additionally, advances in Fc engineering have enhanced the pharmacokinetics of mAbs, reduced immunogenicity, and improved therapeutic efficacy.^50^ These innovations pave the way for next‐generation monoclonal antibody therapies, extending their benefits to previously untreatable conditions.

### 2.7 Biosimilars

mAbs are also subject to innovative challenges, as biosimilar mAbs have gained interest as alternative therapies at lower costs, maintaining the safety and efficacy of the reference mAbs.^51^ For biosimilar mAbs to be approved, they must meet specific standards defined by guidelines, such as those provided by the FDA and the European Medicine Agency (EMA).^51^ These standards state that biosimilars must be highly similar to the reference bioproduct and that there must be no meaningful difference in clinical aspects between them.^52^ The World Health Organization (WHO) also emphasized the importance of the quality and manufacturing process of biosimilar mAbs, highlighting the need for manufacturers to optimize these procedures to minimize differences between the two bioproducts while avoiding any clinical differences.^53^

There are various examples of these biosimilars. In the case of trastuzumab (NCT04784715), a mAb for the treatment of early breast and advanced breast and gastric cancer, up to eight biosimilar mAbs are ready to enter the market as the Roche patent expires.^54^ Another example is adalimumab (NCT00408629), a mAb marketed for treating Crohn's disease and ulcerative colitis.^55^ Before AbbVie's patent expired, three mAbs underwent clinical trials for approval as biosimilars.^56^

The development of biosimilars is a continuous process. Every time a mAb patent is about to expire, other pharmaceutical companies begin developing these molecules and conducting clinical trials for the corresponding approvals.^57^ This phenomenon contributes to reducing the economic burden on healthcare systems globally while providing therapeutic alternatives that preserve the characteristics of the original drug.^58^

## 3 PRODUCTION OF mAbs UNDER GMP CONDITIONS

### 3.1 Large‐scale production of mAbs

The production of mAbs is a complex process that involves bioengineering, industrial bioprocessing, and advanced purification strategies.^59^ In this context, the appropriate methodology must be chosen to ensure high yields and quality for therapeutic use.^11^, ^59^ This section outlines the primary and common steps involved in mAb research, development, and production.

#### 3.1.1 Monoclonal antibodies research and development

##### Classical method: hybridoma technology

**FIGURE 1:**
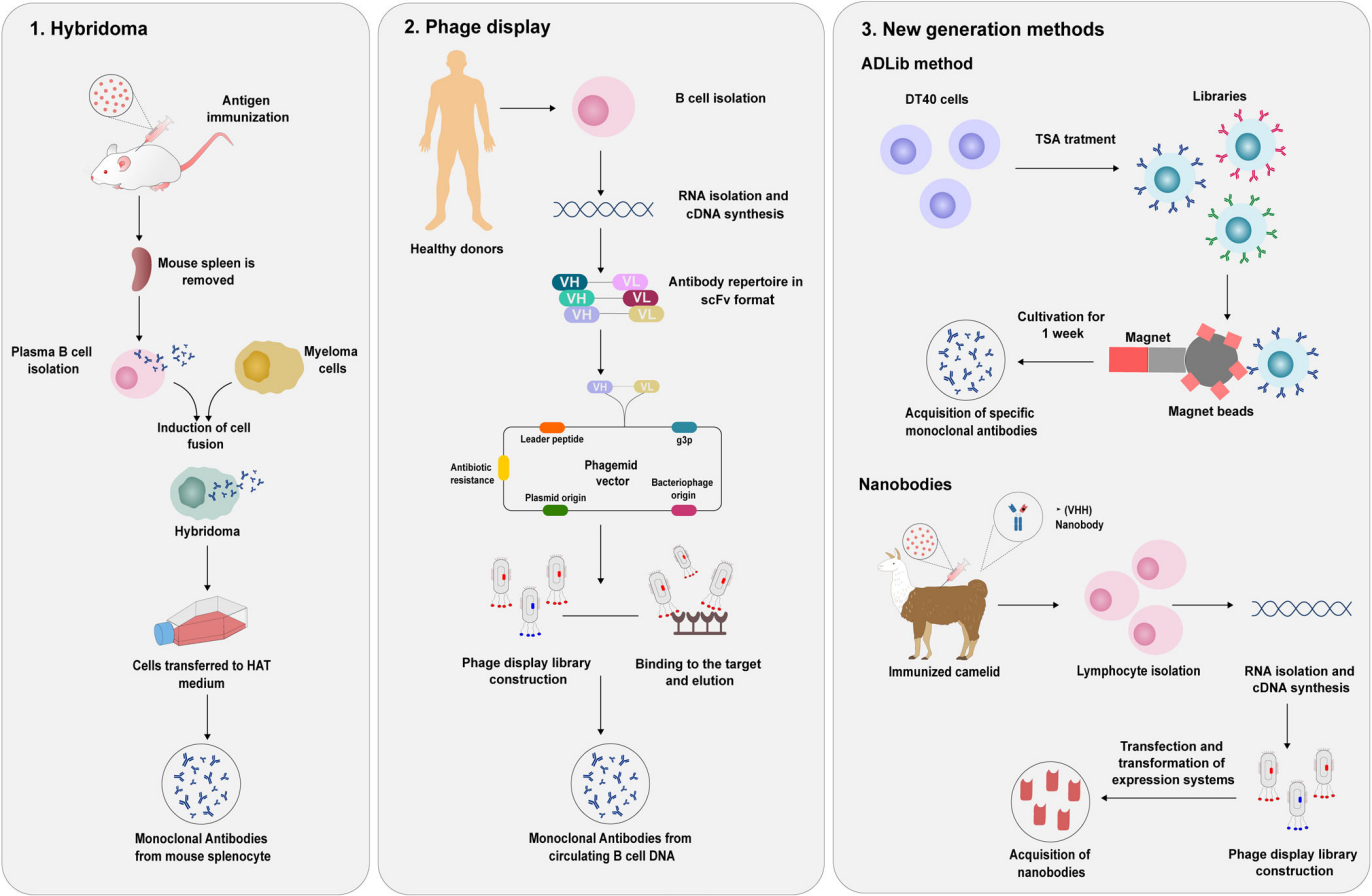
Comparison of hybridoma, phage display, and new‐generation methods for monoclonal antibody (mAb) production. At the laboratory scale, mAbs can be produced via two methods: (1) Hybridoma mAb generation, which begins with immunizing mice with the antigen of interest. The spleen is then harvested to isolate plasma B cells. These plasma B cells are then fused with myeloma cells to create hybridomas that produce mAbs. (2) The phage display methodology relies on antibody genes expressed in filamentous phages, enabling the selection of variants with the highest affinity for the target antigen. This strategy allows the generation of fully human‐compatible antibodies without requiring animal immunization, making it an ethically favorable and efficient method. The process involves isolating mRNA from the B lymphocytes of healthy donors, synthesizing complementary DNA (cDNA) using reverse transcriptase, and amplifying the variable regions of the heavy (V_H_) and light (V_L_) chains using specific primers. The resulting fragments are assembled into single‐chain variable fragments (scFvs), cloned, and inserted into phagemid vectors. They are then amplified in infected bacteria and positively selected by binding to the target antigen for later purification and analysis. (3) New‐generation methods, such as ADLib, utilize DT40 cells derived from avian B cells. This technique involves creating a library that enables the selection of specific antibodies using magnetic beads. On the other hand, nanobodies are derived from heavy‐chain antibodies in camelids immunized with the antigen. These nanobodies are then selected through phage display technology.

The production of mAbs begins with identifying and selecting cell clones capable of secreting specific Abs.^60^ Traditionally, this is achieved through hybridoma technology, which involves the fusion of B cells from mice immunized with an antigen of interest and myeloma cell lines, thereby generating immortalized hybrids that can secrete Abs indefinitely.^61^ These hybrids are maintained in selective culture media, such as hypoxanthine‐aminopterin‐thymidine (HAT) media, to eliminate nonfused cells and obtain productive clones^60^, ^61^ (Figure 1).

One of the primary advantages of this method is its ability to generate mAbs against any antigen of interest.^47^, ^61^ However, this technique has historically faced challenges, including low fusion efficiency and antigen degradation mediated by proteolysis.^61^ Furthermore, mAbs produced in different species carry a risk of disease transmission and immunogenic reactions.^47^

To mitigate these issues, researchers often use humanization technologies, including antibody chimerization, CDR grafting, and human immunoglobulin transgenic rodents, though these approaches can sometimes compromise the mAb's affinity for the antigen.^47^, ^62^

Despite these limitations, hybridoma technology enables the large‐scale production of specific, high‐quality mAbs while preserving the natural pairing information of Abs and the intrinsic capabilities of immune cells.^61^ Recent advances in antibody engineering have overcome species‐level obstacles, enabling the isolation of hybridomas from a variety of phylogenetically diverse species.^63^ Additionally, techniques such as electroporation and electrofusion have been explored to enhance fusion efficiency, offering benefits including higher fusion yields, low cytotoxicity, ease of use, standardization, and greater controllability, all without the use of harmful chemicals or viruses.^47^

##### Selection and expression‐based methods

Alternative methodologies based on phage display technology have been developed.^64^ Here, antibody genes are expressed in filamentous phages, and those with the highest affinity for the target antigen are selected.^65^ This strategy enables the generation of fully human‐compatible Abs without the need for animal immunization, making it an ethically favorable and highly efficient method.^65^

The process begins with the isolation of RNA from B lymphocytes of healthy donors.^64^ Complementary DNA (cDNA) is then synthesized through reverse transcriptase.^64^, ^65^ The genes for the variable regions of the heavy (V_H_) and light (V_L_) chains are subsequently amplified from the cDNA via specific primers that hybridize to the variable domains.^64^ These fragments are assembled to form single‐chain antibody fragments (scFvs), which are then cloned and inserted into a phagemid vector.^65^ Generally, this process results in the construction of a naïve phage library with a diversity ranging from 10^8^ to 10^10^ variants, allowing the selection of Abs with high affinity for their target antigen^64^, ^65^ (Figure 1).

A similar but less commonly used technology is ribosome display, which operates under cell‐free conditions and enables the selection of Abs or high‐affinity proteins by forming an antibody–ribosome–mRNA complex, thereby avoiding product loss during translation.^66^ Briefly, DNA encoding antibody variants is transcribed and translated without a stop codon, allowing the protein to remain attached to the ribosome and mRNA.^67^

Then, the target antigen is included, and high‐affinity complexes are selected and recovered. These complexes are converted to cDNA via RT‐PCR and amplified for subsequent selection cycles.^47^ This system can evaluate large libraries (10^12^–10^15^ variants) in a single reaction, enabling rapid identification of high‐affinity antibody fragments.^47^ Its advantages include the absence of a cellular culture and the ability to perform post‐translational modifications in eukaryotic systems.^67^

Finally, synthetic and semi‐synthetic phage‐display libraries, designed on human germline frameworks with diverse CDRs, provide predefined sequence diversity and enable iterative in vitro affinity maturation.^68^ These libraries reduce the need for extensive downstream engineering by generating variants with improved biophysical properties and higher initial specificity than naive or non‐designed libraries.^69^

##### New‐generation methods

On the other hand, new technologies have emerged for the rapid generation of specific mAbs, avoiding the use of animals and generating genetically stable clones.^47^ One of the most relevant systems is ADLib, which uses avian B cells (DT40) that can diversify their immunoglobulin genes via genetic conversion (Figure 1).^70^ Recently, this technology has been improved by incorporating a molecular control mechanism for the Activation‐Induced Deaminase (AID) enzyme, which initiates this diversification through an auxin‐inducible degradation system (AID^2^ system).^70^ This method allows activation of AID to generate diversity during library construction, followed by deactivation to halt genetic conversion, thereby maintaining the stability of the selected clones. This new technology enables the production of mAbs in a few days with high affinity and without the need for animal use, offering an efficient, controllable, and reproducible platform for the development of mAbs.^70^

Recent investigations have developed Abs composed solely of heavy chains found in camelids, such as llamas and alpacas (Figure 1).^71^ These Abs enable the isolation and expression of variable domains, known as nanobodies (Nbs) or single‐domain Abs.^72^ Nbs can be sourced from immune, naïve, or synthetic libraries and selected using techniques such as phage display and resonance. Today, Nbs are used in various applications, including cancer therapy, treatment of central nervous system diseases, infection management, and immunotherapy.^8^ Additionally, these pharmaceuticals can be produced in goats, llamas, and alpacas.

#### 3.1.2 Master Cell Bank, Working Cell Bank, and monoclonality assurance

To ensure GMP‐compliant monoclonal antibody production, it is crucial to establish well‐characterized Master Cell and Working Cell Banks (MCB and WCB).^73^ The MCB consists of a homogeneous population of a single, stable producer clone.^74^ This clone is expanded under controlled conditions and cryopreserved in multiple vials, which serve as the primary reference stock for manufacturing.^74^ The WCB is prepared by expanding one or more MCB vials to generate sufficient material for routine production.^74^ This two‐tiered banking system effectively minimizes genetic drift, ensures batch‐to‐batch consistency, and supports a long‐term supply in accordance with GMP requirements.^75^

Regulatory guidelines require a comprehensive characterization of both the MCB and WCB. This includes tests for sterility, mycoplasma, adventitious viruses, identity, genetic stability, productivity, and cell‐line authentication using molecular or biochemical methods.^73^, ^75^, ^76^

A critical GMP requirement is demonstrating monoclonality in the production cell line.^74^, ^75^ Monoclonality confirms that the final manufacturing process is derived from a single progenitor cell, thereby minimizing heterogeneity and reducing the risk of genetic or phenotypic variation.^73^, ^74^, ^75^ Current regulatory expectations include image‐based evidence of monoclonality obtained during single‐cell cloning procedures, such as limiting dilution, semi‐automated cloning, or fluorescence‐activated cell sorting (FACS) for single‐cell deposition, which facilitates high‐confidence verification of monoclonality in compliance with ICH Q5D and EMA guidelines.^77^

#### 3.1.3 Antibody production and quality control under GMP conditions

##### Recombinant expression in mammalian cells

The genes encoding the antibody V_H_ and V_L_ chains are inserted into expression vectors and transferred into suitable cell lines for high‐yield protein production.^8^ The most frequently used cell lines for this purpose in the biotechnology industry include Chinese hamster ovary (CHO) cells, human embryonic kidney (HEK293) cells, and murine myeloma (NS0) cells.^8^, ^78^ CHO cells are widely preferred due to their ability to perform human‐like post‐translational modifications, their high productivity, and their capacity for suspension growth.^60^

**FIGURE 2:**
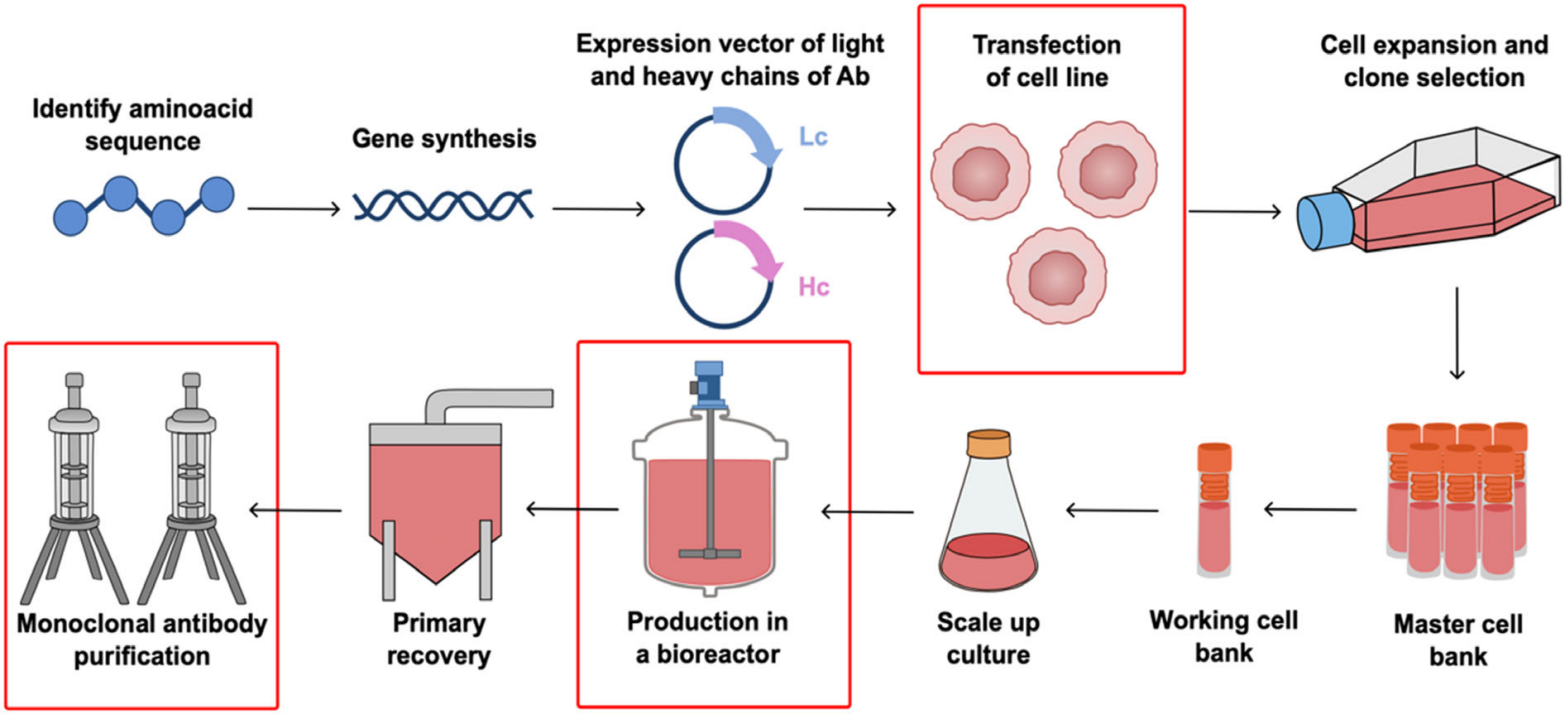
Large‐scale good manufacturing practices‐based monoclonal antibodies (mAb) production. mAb production begins with the identification of the amino acid sequence of the mAb of interest and the synthesis of the corresponding ORF and gene, which is inserted into a eukaryotic expression vector. Cell lines such as Chinese hamster ovary, HEK293, or NS0 are commonly used, which are transfected, expanded, and selected to establish a master cell bank, followed by working cell banks for each batch. Cells are cultured in increasingly larger formats and bioreactors, followed by primary recovery of mAbs to remove cell debris and purification steps to ensure the quality of the final product. Strict traceability and quality controls are implemented throughout this process to ensure batch‐to‐batch consistency, safety, and compliance with relevant regulations. Critical steps in the process are highlighted with red squares, including (i) cell transfection, which is influenced by transfection efficiency; (ii) bioreactor production, which depends on critical parameters such as temperature, agitation, and pH; and (iii) mAb purification, a crucial step that directly impacts the final product's quality.

The generation of stable cell lines involves the transfection of plasmid vectors containing antibody genes and selection genes, including metabolic selectable markers such as dihydrofolate reductase (DHFR) and glutamine synthase (GS), or antibiotic selectable markers such as puromycin acetyltransferase and blasticidin deaminase.^8^, ^60^ Selective pressure is applied to obtain clones that express high levels of Abs, which are optimized through gene amplification and clonal selection techniques^8^ (Figure 2).

##### Cell culture and large‐scale production

Once the producing cell line has been established, it must be expanded in a cell bank to scale up mAb production^60^ (Figure 2). To this end, it is crucial to scale up production by transitioning from shake flasks to bench‐top bioreactors.^79^ Pilot‐scale production plays a crucial role in bridging the gap between laboratory research and industrial applications. It enables the evaluation of critical engineering parameters, including oxygen transfer rate (kLa), power input per volume (*P*/*V*), mixing time, gas–liquid mass transfer, and shear forces. These parameters must be maintained within ranges that support cell growth and the quality of the final product.^59^

Critical process parameters (CPPs) can be grouped into physical–chemical and culture‐related variables. Physical–chemical variables include agitation rate (rpm), temperature, pH, dissolved oxygen (DO), osmolarity, gas composition, and when applicable, operating pressure.^80^ Culture‐related variables encompass the basal medium formulation, feeding strategy (bolus, exponential, or continuous feeding), viable cell density, specific productivity (qP), and key metabolites such as glucose, glutamine, lactate, and ammonium.^81^ Importantly, metabolites such as lactate and ammonium are not completely removed from the culture but are instead maintained within acceptable ranges through optimized feeding strategies, pH control, medium design, and in some cases, perfusion‐based exchange.^82^

Various culture strategies can be employed, including batch (closed culture), feed‐batch (progressive feeding of nutrients), and perfusion (continuous extraction of the product while the cells continue to grow).^60^, ^83^ Perfusion culture is particularly attractive for mAb production, as it enables higher cell density and continuous antibody production with reduced variability in quality.^59^, ^60^, ^84^ Perfusion systems not only enable the maintenance of a continuous supply of mAbs during the upstream phase but also control the accumulation of toxic byproducts, such as ammonium or lactate, during culture.^84^

On the other hand, fed‐batch cultures require formulating the culture medium for feeding and optimizing nutrient supply strategies.^85^ To this end, techniques such as titrating individual components, analyzing the medium, and mixing media are used to enhance growth.^85^ Due to the sanitary risk, it is essential that the medium used not contain animal components, such as fetal bovine serum. Instead, it is important to opt for chemically defined media that contain amino acids, vitamins, salts, and growth factors.

Bioprocess control is essential for maintaining culture stability and maximizing productivity.^60^ Critical variables such as pH, DO concentration, osmolality, and nutrient concentration must be continuously monitored and adjusted.^59^ CPPs must be continuously monitored and controlled to ensure process stability and consistent results and product quality.^86^ During culture in bioreactors, CPPs play a crucial role in the final product of the upstream process; therefore, establishing a robust quality by design (QbD) framework is essential to ensure the quality and efficiency of the process.^87^

Tank dimensions and impeller types also significantly affect the hydrodynamics of cell cultures; therefore, the initial setup of the bioreactors must be meticulously studied before implementation.^88^ The need to improve the design of biopharmaceutical processes has prompted institutions to collaborate to develop a more efficient approach to developing scalable production platforms.^89^

##### Antibody purification and characterization

mAb purification is crucial for eliminating impurities and guaranteeing a high‐quality final product (Figure 2).^8^ The most commonly used purification strategy is based on protein A‐coupled affinity chromatography, where Abs bind specifically to protein A from *Staphylococcus aureus* (SpA), which is produced recombinantly.^90^ Alternatively, protein G from *Streptococcus* spp. can be used, depending on the isotype of the antibody being purified.^91^ Protein A or protein G is immobilized on a solid matrix, allowing the separation of mAb from the culture medium.^59^, ^92^ Subsequently, ion exchange and size‐exclusion chromatography remove protein aggregates and unwanted protein fragments.^92^ Other types of purification using different proteins could be performed, as shown in Table 1.^91^

**TABLE 1:** Proteins commonly used for monoclonal antibodies purification.^91^

Protein	Source	Binding specificity
Protein A	*Staphylococcus aureus*	Fc region
Protein G	*Streptococcus* spp.	Fc region
Protein L	*Peptococcus magnus*	κ light chains
Protein D	*Branhamella catarrhalis*	IgD
Protein P	*Clostridium perdringens*	κ light chains

To ensure product safety, viral filtrations are applied to eliminate possible biological contaminants via gel filtration chromatography (GFC) or hydrophobic interaction chromatography (HIC), depending on the molecule and its properties.^8^, ^93^ Additionally, ultrafiltration and diafiltration enable the concentration and adjustment of Abs for their final formulation.^92^

Antibody characterization to ensure purity is performed using advanced analytical techniques, including mass spectrometry to determine molecular mass, high‐pressure liquid chromatography (HPLC) to assess purity, surface plasmon resonance (SPR) to measure antigen binding affinity, and biological assays to validate function.^92^

##### Formulation and storage

The final step in producing mAbs is their formulation to ensure stability and efficacy during storage and administration.^94^ Excipients such as sugars, amino acids, and surfactants are added to prevent antibody aggregation and denaturation.^8^, ^60^ Depending on the final product, mAbs can be stored as liquid formulations at 2–8°C, as frozen bulk at temperatures of −20°C or below, or in lyophilized form to enhance long‐term stability.^8^, ^60^, ^89^ Freeze–thaw cycles and temperature fluctuations can induce aggregation and structural changes, potentially affecting product quality and efficacy. As a result, controlling the cold chain, both before and after marketing, is a critical GMP requirement. This ensures that therapeutic Abs remain stable, functional, and safe throughout their storage, distribution, and clinical use.^8^, ^60^, ^94^

### 3.2 GMP standards for mAb production

The application of GMP is essential to ensure that pharmaceutical products, including mAbs, are consistently produced and controlled according to stringent quality standards. The production of mAbs involves complex biological processes that require meticulous oversight to mitigate risks such as contamination, variability, and inconsistency between batches.^59^ The WHO emphasizes that GMP ensures the safety and efficacy of biologics, facilitating international regulatory alignment and streamlining the approval and distribution of these critical therapeutic products.^95^, ^96^

Recent regulatory updates have strengthened control of aseptic operations in mAb manufacturing. The 2022 revision of the EU GMP annex 1 introduced stricter requirements for contamination control strategies, environmental monitoring, and facility design. One key addition is the mandatory use of process simulation tests, known as media fills, in which a nutrient medium replaces the actual product to closely replicate all steps of aseptic manufacturing. Manufacturers must successfully complete three consecutive simulations to demonstrate the robustness of their aseptic processes.

Annex 1 further underscores the importance of cleanroom classification and airflow management, ensuring compliance with ISO 14644‐1, which governs particulate concentration limits for grades A–D environments. These updates emphasize that facility design, heating, ventilation, and air conditioning (HVAC) performance, and sterility assurance systems are essential components of GMP, designed to minimize the risk of microbiological contamination throughout the antibody production process.

Recent advancements, such as QbD approaches, have refined mAb production by integrating predictive models and analytics to increase consistency and scalability.^97^ Moreover, innovations in cell line engineering and bioreactor technology, including fed‐batch and perfusion systems, have enabled higher yields of mAb production and reduced production costs, making mAbs more accessible for a broader range of therapeutic applications.^60^ The WHO guidelines further stress the importance of robust environmental monitoring systems within GMP facilities, ensuring compliance with cleanliness standards and minimizing the risk of microbial contamination.^96^ Similarly, the EMA guidelines emphasize the crucial role of risk‐based cleaning validation protocols in preventing cross‐contamination, particularly in multiproduct facilities.^98^

The successful implementation of GMP in mAb manufacturing relies on the integration of multiple core components that safeguard product quality and patient safety.^95^, ^96^ A robust pharmaceutical quality system (PQS) is fundamental, providing a structured framework to align all manufacturing activities with regulatory standards and fostering continuous improvement.^97^ This system emphasizes risk‐based decision‐making to address inherent variabilities in biological production.^97^, ^99^

Equally important is the comprehensive training of personnel in microbiology, biochemistry, and GMP protocols, ensuring that they possess the necessary skills to handle sensitive biologics and maintain aseptic conditions throughout the production lifecycle.^95^, ^96^, ^98^

Manufacturing facilities and equipment must adhere to strict design and operational standards.^8^ For example, HVAC systems are critical for maintaining controlled environments, and all equipment must undergo rigorous qualifications and validation processes to ensure consistent performance.^100^ Comprehensive documentation underpins GMP compliance by providing traceability and accountability, with detailed records such as batch production logs and validation protocols facilitating audits and regulatory inspections.^97^, ^100^

Throughout the development and manufacturing of mAbs, the scope of GMP requirements gradually increases from early discovery to clinical production. While screening and initial engineering activities are not performed under GMP conditions, the establishment of monoclonal producer cell lines, MCB/WCB generation, and subsequent upstream and downstream processes fall under the framework of EU GMP annex 1 (aseptic processing, sterile filtration, filling, and storage) and annex 2 (biological products, from API production to batch release). In practice, GMP‐compliant mAb manufacturing faces several challenges, including high production costs, particularly for chemically defined media, perfusion systems, and Protein A chromatography.^89^ These challenges also include control of long‐term cell line stability, facility design and contamination control strategies, and the complexity of scaling up from small research and development (R&D) reactors to industrial bioreactors. Additional challenges arise when development or manufacturing is outsourced to CDMOs, requiring strict oversight to maintain GMP alignment.^101^ Finally, innovative technologies such as CRISPR‐based cell engineering, G‐Rex culture platforms, continuous purification approaches (e.g., cFAE), and plant‐based expression systems offer promising opportunities; however, their integration into GMP workflows requires rigorous validation and regulatory assessment.

Additionally, real‐time process control ensures the monitoring of critical quality attributes (CQAs), such as pH, temperature, and DO levels, thereby ensuring consistency during key manufacturing steps, including cell culture and purification.^60^, ^99^ Proactive risk management further enhances GMP adherence by identifying and mitigating potential issues, including contamination with adventitious agents, through an adequate process of environmental control and monitoring.^102^ Overall, the production of mAbs must be as rigorous as possible for their use as therapeutics. Hence, QbD is of critical importance during the development process.

It is also essential to consider that stages such as the humanization of mAbs must undergo specific steps to reduce immunogenicity reactions, which aligns with the GMP guidelines for biologics.^95^, ^96^ Additionally, the WHO developed a guideline for the nonclinical and clinical evaluation of mAbs and related products intended to prevent or treat infectious diseases, describing the correct path for testing novel therapeutic mAbs.^96^

## 4 VALIDATION AND TRACEABILITY SYSTEMS

### 4.1 Validation of mAb production processes under GMP conditions

The validation of production processes for mAbs is a cornerstone of GMP compliance, ensuring that each step consistently meets predefined criteria.^103^ Process validation involves rigorous testing to demonstrate that all stages of mAb production, from upstream cell culture to downstream purification processes, operate within established parameters, ensuring product integrity and reproducibility.^53^

According to the WHO guidelines, facility design and process flow are crucial for successful validation, with particular attention given to separating clean and unclean zones to minimize the risk.^96^ HVAC systems must be validated to maintain appropriate air pressure differentials and filtration efficiency, particularly in areas where aseptic processing occurs.^53^, ^95^ The EMA guidelines also highlight the importance of validating cleaning procedures to ensure that equipment surfaces meet stringent cleanliness criteria, which are required to prevent the accumulation of residues and cross‐contaminants that may compromise product quality.^98^ This includes the design of robust process performance qualification (PPQ) protocols that evaluate process consistency over consecutive production runs.^95^

Equipment validation is equally critical and requires thorough initial and periodic testing to confirm the reliable performance of both tools and equipment, such as bioreactors, chromatographic systems, and sterilization units.^95^, ^96^ These validations must address the cleaning and maintenance status of equipment to prevent contamination and cross‐contamination, especially in multiproduct facilities, as well as their performance.^95^

Analytical validation ensures that methods used to assess product quality, including potency assays, purity assessments, and stability studies, are robust, reproducible, and capable of detecting the presence of process‐related impurities, thereby ensuring that the product conforms to stringent specifications.^104^ Special emphasis is placed on viral clearance and the removal of host cell DNA and proteins during downstream purification steps to comply with safety standards.^105^, ^106^ Validation protocols must also demonstrate the scalability of production processes, ensuring that laboratory‐scale data are transferable to full‐scale manufacturing.^98^ These validations collectively reinforce the reliability of the manufacturing process, aligning with stringent regulatory requirements and therapeutic standards.^95^, ^96^

### 4.2 Traceability during the production of mAbs

Traceability in the production of mAbs is crucial to ensure quality, regulatory compliance, and patient safety.^107^ A robust traceability system allows multiple inputs from equipment, product batches used in the manufacturing process, and all performed procedures and measurements during production to be tracked, ensuring data recording for traceability and transparency in the process, from the supply chain to the final product, facilitating the rapid identification of quality issues.^107^ Furthermore, it enables the efficient detection and recall of defective and falsified batches, minimizing patient risks and optimizing operational costs.^108^

Data collected throughout the manufacturing process must comply with data‐integrity principles to ensure its reliability and regulatory acceptability.^109^ These principles are summarized by the ALCOA framework, which requires that data be Attributable, Legible, contemporaneously recorded, Original (or an approved true copy), and Accurate. Adherence to ALCOA ensures that all information generated during mAb production is trustworthy and supports the safe and consistent use of the final product.^101^ Consequently, data integrity is a fundamental requirement that complements traceability across the entire manufacturing process.^109^

Blockchain technology has emerged as a key solution to improve drug traceability.^110^ Blockchain allows transactions to be recorded in a decentralized, immutable, and secure manner, significantly reducing the risk of fraud and counterfeiting.^111^ Through a distributed database, each transaction or movement of a batch of mAbs is recorded in a block, validated, and added to the chain, making it impossible to modify without altering the entire data structure.^110^, ^111^, ^112^ This technology also allows integration with advanced identification systems, such as quick response (QR) codes, radio frequency identification (RFID) tags, and Internet of Things (IoT) sensors, facilitating real‐time authentication of pharmaceutical products throughout the distribution chain.^113^

One of the main challenges in implementing blockchain in the biopharmaceutical industry is the need for standardization of traceability data and interoperability between different actors in the supply chain.^103^ Similarly, the transition from traditional paper‐based traceability systems to digital platforms involves implementation costs, technological infrastructure, and staff training challenges. However, potential benefits include reduced administrative costs, improved regulatory efficiency, guaranteed product authenticity, and decreased circulation of counterfeit mAbs.^114^

## 5 QUALITY CONTROL

QC is a fundamental component of GMP, ensuring that every batch of mAbs meets rigorous safety, purity, and potency standards before being released for therapeutic use.^53^ Facilities must adhere to strict environmental and process control protocols.^53^, ^95^ The WHO guidelines emphasize the importance of cleanroom classification and adherence to controlled environmental standards to minimize the risk.^95^ Regularly monitoring particulate and microbial loads and appropriate gowning and personnel movement protocols are critical for maintaining aseptic conditions.^96^ The EMA guidelines complement this aspect by outlining requirements for HVAC systems, air filtration, and pressure differentials to prevent cross‐contamination between manufacturing zones, particularly in multiproduct facilities.^98^

QC involves a comprehensive suite of activities, including detailed analytical testing, microbiological assessment, and stability monitoring to ensure product quality throughout its lifecycle.^115^ The WHO guidelines emphasize the role of advanced environmental monitoring systems in maintaining aseptic conditions within cleanrooms, using both active air sampling and settling plates to track microbial contamination.^96^ The EMA guidelines further underscore the necessity of implementing robust validation protocols for HVAC systems, ensuring proper airflow direction, pressure differentials, and particulate filtration to prevent cross‐contamination.^98^

Additionally, frequent reassessment of facility layouts and operational processes is recommended to adapt to new products or process changes, reducing contamination risk and increasing compliance with evolving regulatory standards.^53^, ^95^, ^96^ Testing confirms the specific molecular and structural characteristics of the mAb via techniques such as peptide mapping, mass spectrometry, and nuclear magnetic resonance (NMR) spectroscopy.^53^, ^116^ Purity and impurity analysis uses HPLC, capillary electrophoresis, and size‐exclusion chromatography to detect and quantify host cell proteins, DNA residues, and aggregates, ensuring that contaminants remain below acceptable thresholds.^117^ Potency assays are conducted via cell‐based bioassays and enzyme‐linked immunosorbent assays (ELISAs) to validate the ability of an mAb to achieve its therapeutic mechanism of action, such as binding to target antigens or eliciting immune responses, such as antibody‐dependent cellular cytotoxicity (ADCC).^118^

Microbiological testing ensures sterility and endotoxin detection, with advanced rapid microbiological methods supplementing traditional culture techniques to reduce testing timelines.^119^ Stability studies evaluate the long‐term and accelerated stability of mAbs under various storage conditions, assessing critical parameters such as temperature sensitivity, aggregation, and degradation.^53^, ^119^

Furthermore, QC employs statistical tools for continuous monitoring and trend analysis to address product variability, identifying shifts or trends in product quality attributes across multiple batches.^53^ This proactive approach enables early detection and resolution of potential process deviations, enhancing product reliability.^120^ QC protocols are designed to align with the principles of QbD, emphasizing a thorough understanding of the relationship between CQAs and process parameters.^106^ The role of QC extends beyond initial batch testing to include ongoing oversight during postmarketing surveillance, ensuring that the therapeutic efficacy and safety of mAbs are maintained throughout their commercial lifecycle.^53^, ^106^, ^118^, ^120^

## 6 CURRENT CHALLENGES IN THE GMP PRODUCTION OF mAbs

As the global demand for mAbs continues to rise, the biopharmaceutical industry faces multiple challenges in implementing GMP standards for mAb production.^105^ These include bioprocess optimization, cost reduction, scalability improvement, and adaptation to ever‐evolving regulatory norms.^53^, ^100^, ^105^ Furthermore, integrating new technologies and establishing a secure and efficient supply chain are key factors in ensuring the quality and availability of these biotherapeutics.^105^

Full‐length therapeutic mAbs require mammalian cell expression systems for production, as these platforms allow the generation of biologically functional proteins with essential post‐translational modifications.^47^ However, in the case of mAb fragments and recombinant Fs with simplified or no glycosylations, it is possible to employ expression systems in lower organisms, such as bacteria or yeast.^121^

Unlike oligopeptides, which can be chemically synthesized, mAbs are large complex structures (monomers of 140–160 kDa) composed of four polypeptide chains (two heavy chains and two light chains) linked by disulfide and noncovalent bonds.^121^, ^122^ They also exhibit conserved glycosylation in the Fc region (N297), which is crucial for their stability and immune effector functions.^123^

The development, manufacturing, and formulation of mAbs for therapeutic purposes present significant challenges that have driven the search for innovative strategies to optimize their production and clinical efficacy.^121^ Like other biotherapeutics, mAbs rely on cell‐based expression systems, making them costly and inefficient compared with other products obtained by chemical synthesis, such as small molecules. Variability in yields, depending on the product and expression system used, represents another challenge, as it is required for subsequent processes to remove biological contaminants from the production system.^121^

While affinity chromatography is a robust technology for the initial capture of mAbs during their purification, subsequent steps, such as viral inactivation, can expose the product to extreme pH and salt concentration conditions.^117^ These conditions can induce chemical degradation of the mAb, affecting its stability and decreasing the recovery of the final product.^121^

The implementation of technological tools, such as real‐time data management systems and automation in manufacturing processes, has gained importance in the biopharmaceutical industry.^14^ New regulations and standards are constantly emerging when mAbs are produced. Therefore, adapting manufacturing procedures, documentation, and QCs to ensure continuity in production in compliance with requirements is crucial.^2^, ^4^

The supply chain involves significant challenges due to the need for complete traceability and rigorous input control, which ensure compliance with current regulations.^105^, ^106^ Material sensitivity, adulteration risks, and logistical disruptions further complicate its management.

A reliable supply chain is essential to minimize risks and ensure continuity in drug manufacturing, highlighting the need for robust control and planning systems.^2^, ^4^ The production of mAbs following GMP standards faces significant challenges, such as product structural complexity, high production costs, stringent regulatory standards, and batch‐to‐batch variability. In addition, one of the greatest challenges is the efficient transition from R&D phases to industrial‐scale production.^2^, ^13^

## 7 CONCLUSIONS

The production of mAbs under GMP standards is a highly regulated and technically demanding process, where each stage, from the selection and optimization of cell lines to purification and final formulation, must guarantee the product's quality, safety, and efficacy. The implementation of rigorous validation systems and traceability throughout the process has enabled the improvement of reproducibility and the minimization of risks associated with contamination and variability between batches.

However, significant challenges persist, including high production costs, the need to develop strategies to enhance the efficiency of bioprocesses, and the ongoing need to update regulations in response to new technologies and therapeutic approaches.

Advances in biotechnology have driven the development of innovative tools, including the optimization of cell cultures through artificial intelligence, the use of continuous bioprocesses, and the incorporation of real‐time tracking technologies. In addition, blockchain applications in drug traceability offer unprecedented potential to enhance security and transparency in the supply chain. However, implementing these technologies requires overcoming regulatory and economic barriers, highlighting the need for a flexible regulatory framework that fosters innovation without compromising quality.

Overall, mAb production needs to balance growing demand with the sustainability and accessibility of these treatments. Harmonizing international standards, optimizing manufacturing processes, and integrating digital tools will ensure efficient and accessible production globally. Only through a combination of technological advances and dynamic regulations will the therapeutic impact of mAbs in various areas of medicine continue to expand.

## AUTHOR CONTRIBUTIONS

**TT‐B**: conceptualization, writing the original draft, writing the initial draft, review, editing, figure design, and revision of all versions; **VP**: writing the initial draft, revision of original draft, editing; **PL**: writing the initial draft, revision of original draft, revision of final version; **JDR**: writing the initial draft, revision of original draft, revision of final version; **CA**: writing the initial draft, revision of original draft, revision of final version; **HFP**: revision of original draft and revision of final version; **SMB**: revision of original draft and revision of final version; **PAG**: revision of original draft and revision of final version; **AMK**: conceptualization, revision of original draft, editing, and revision of final version. All the authors contributed to the article and approved the submitted version.

## FUNDING INFORMATION

This study was supported by ANID “Fondo Nacional de Ciencia y Tecnología de Chile” (Fondecyt) Regular 1231851 (Alexis M. Kalergis), 1231905 (Susan M. Bueno), 1240971 (Pablo A. González), Fondecyt de Iniciación 11230573 (Hernán F. Peñaloza), and the Millennium Institute on Immunology and Immunotherapy, ANID—Millennium Science Initiative Program ICN2021_045 (former P09/016‐F, ICN09_016), BMRC P7 (Alexis M. Kalergis, Susan M. Bueno).

## CONFLICT OF INTEREST STATEMENT

The authors declare that they have no conflicts of interest.

## Data Availability

Data sharing not applicable to this article as no datasets were generated or analysed during the current study.
